# Cost-effectiveness of Physical Therapy vs Intra-articular Glucocorticoid Injection for Knee Osteoarthritis: A Secondary Analysis From a Randomized Clinical Trial

**DOI:** 10.1001/jamanetworkopen.2021.42709

**Published:** 2022-01-24

**Authors:** Daniel I. Rhon, Minchul Kim, Carl V. Asche, Stephen C. Allison, Chris S. Allen, Gail D. Deyle

**Affiliations:** 1 Department of Rehabilitation Medicine, Brooke Army Medical Center, JBSA Fort Sam Houston, Texas; 2 Department of Rehabilitation Medicine, Uniformed Services University of Health Sciences, Bethesda, Maryland; 3 Department of Internal Medicine, University of Illinois College of Medicine at Peoria, Peoria; 4 Department of Rehabilitation, College of Allied Health Sciences, University of Cincinnati, Cincinnati, Ohio

## Abstract

**Importance:**

Physical therapy and glucocorticoid injections are initial treatment options for knee osteoarthritis, but available data indicate that most patients receive one or the other, suggesting they may be competing interventions. The initial cost difference for treatment can be substantial, with physical therapy often being more expensive at the outset, and cost-effectiveness analysis can aid patients and clinicians in making decisions.

**Objective:**

To investigate the incremental cost-effectiveness between physical therapy and intra-articular glucocorticoid injection as initial treatment strategies for knee osteoarthritis.

**Design, Setting, and Participants:**

This economic evaluation is a secondary analysis of a randomized clinical trial performed from October 1, 2012, to May 4, 2017. Health economists were blinded to study outcomes and treatment allocation. A randomized sample of patients seen in primary care and physical therapy clinics with a radiographically confirmed diagnosis of knee osteoarthritis were evaluated from the clinical trial with 96.2% follow-up at 1 year.

**Interventions:**

Physical therapy or glucocorticoid injection

**Main Outcomes and Measures:**

The main outcome was incremental cost-effectiveness between 2 alternative treatments. Acceptability curves of bootstrapped incremental cost-effectiveness ratios (ICERs) were used to identify the proportion of ICERs under the specific willingness-to-pay level ($50 000-$100 000). Health care system costs (total and knee related) and health-related quality-of-life based on quality-adjusted life-years (QALYs) were obtained.

**Results:**

A total of 156 participants (mean [SD] age, 56.1 [8.7] years; 81 [51.9%] male) were randomized 1:1 and followed up for 1 year. Mean (SD) 1-year knee-related medical costs were $2113 ($4224) in the glucocorticoid injection group and $2131 ($1015) in the physical therapy group. The mean difference in QALY significantly favored physical therapy at 1 year (0.076; 95% CI, 0.02-0.126; *P* = .003). Physical therapy was the more cost-effective intervention, with an ICER of $8103 for knee-related medical costs, with a 99.2% probability that results fall below the willingness-to-pay threshold of $100 000.

**Conclusions and Relevance:**

A course of physical therapy was cost-effective compared with a course of glucocorticoid injections for patients with knee osteoarthritis. These results suggest that, although the initial cost of delivering physical therapy may be higher than an initial course of glucocorticoid injections, 1-year total knee-related costs are equivalent, and greater improvement in QALYs may justify the initial higher costs.

**Trial Registration:**

ClinicalTrials.gov Identifier: NCT01427153

## Key Points

**Question:**

Is an initial treatment of physical therapy cost-effective compared with an initial treatment of glucocorticoid injection for patients with knee osteoarthritis?

**Findings:**

In this economic evaluation that included 156 adults participating in a randomized clinical trial, participants receiving physical therapy gained more quality-adjusted life-years compared with those receiving glucocorticoid injection. Costs related to knee care were similar between groups, although total medical costs for any reason were higher in the physical therapy group.

**Meaning:**

The results of this study suggest that although the mean cost of providing an initial course of physical therapy may be higher than an initial course of glucocorticoid injections, the greater improvement in quality-adjusted life-years may be worth the additional cost.

## Teaser

This economic evaluation compares the cost-effectiveness of physical therapy vs intra-articular glucocorticoid injection as initial treatment strategies for knee osteoarthritis.

## Introduction

Osteoarthritis is the most common type of arthritis, accounting for a disproportionate level of disability and health care expenditures worldwide. It is the eighth most expensive of 154 health conditions to manage in the US, costing more than $80 billion in 2016.^1^ The knee is one of the more common joints affected by osteoarthritis, with its prevalence doubling during the last 70 years.^2^ Knee osteoarthritis may begin early in life and progresses slowly, providing opportunities for nonsurgical treatment options.^3^

Effective nonsurgical treatment options are available for knee OA that should be considered before surgery. The American College of Rheumatology provides guidelines that include 6 strongly and 8 conditionally recommended nonpharmacological interventions, as well as 3 strongly and 4 conditionally recommended pharmacological treatments.^4^ With so many choices, patients and clinicians often find it challenging to establish optimal treatment plans. One critical component for decision-making is the cost and accessibility of the intervention.

Physical therapy and intra-articular glucocorticoid injections are common treatments for knee osteoarthritis. Data from large health care systems suggest that they are infrequently delivered together,^5,6^ indicating that patients or their practitioners are more likely to choose one or the other. Exercise therapy, a core nonpharmacological intervention for knee osteoarthritis, is a staple of physical therapy programs. Manual therapy also has proven to be an effective treatment in several clinical trials.^7,8,9^ A single glucocorticoid injection typically delivered during the same consultation with the diagnosing clinician may be initially less expensive and less time intensive than a course of physical therapy. However, there is concern that glucocorticoid injections may accelerate degenerative changes in the longer term and may not add to the benefit of physical therapy in the short term.^10,11,12,13^ Consideration for both short- and longer-term outcomes, treatment risks and adverse effects, and the downstream effect of each treatment choice should influence and inform these decisions instead of initial cost alone. Value is defined by the cost incurred to generate a change in outcome.^14^ Some interventions with lower initial costs may not carry that cost-effectiveness into the long term. Although physical therapy was clinically more effective than glucocorticoid injection in a recent trial,^15^ a typical course is usually more costly than a single injection ($99-$172 for a primary care office visit and injection procedure and $557-$919 for a low-complexity physical therapy evaluation and 7 additional physical therapy sessions consisting of manual therapy and 30 minutes of exercise per the 2020 Medicare Physician Fee Schedule).^16^ Given the high prevalence of knee osteoarthritis and the frequency of practical decisions made about best treatment options, we aimed to assess the cost-effectiveness of physical therapy compared with glucocorticoid injection from the health care perspective.

## Methods

### Overview

We conducted an economic evaluation using patient-level data from a randomized clinical trial^15^ that captured health care system costs, including knee-specific care costs, and quality-adjusted life-years (QALYs), which is a 2-dimensional metric that incorporates both health-related quality of life and survival. We used the Panel on Cost-Effectiveness in Health and Medicine guidelines for conducting the cost-effectiveness studies^17,18^ as well as the Consolidated Health Economic Evaluation Reporting Standards (CHEERS) reporting guideline^19^ to guide reporting of these results. Although we noted a priori that we would capture health care use and compare it between groups in our published protocol,^20^ we did not formally establish a statistical plan for the cost-effectiveness analysis at that time. Therefore, this evaluation should be considered an unplanned secondary analysis. The health economist and team of statisticians were blinded to the results of the primary study, and the original publication was not published before they planned and performed these analyses. The statistical analysis plan was developed and finalized before receipt of any data. This case analysis comes from the health care perspective rather than payer or societal perspective. The health care perspective includes both current and future costs of care, both related and unrelated to the condition.^21^ The exception is that out-of-pocket costs were not assessed; however, there is no copayment for care in military treatment facilities, making this potentially less relevant. All participants provided written informed consent. All data were deidentified. Ethics approval was granted for the trial by the Army Pacific Health Command Institutional Review Board.

### Data

The full results of the 1-year randomized clinical trial were previously reported.^15^ Briefly, we recruited patients who met clinical and radiographic criteria for the presence of knee osteoarthritis established by the American College of Rheumatology^21^ from October 1, 2012, to May 4, 2017, at 2 large military hospitals in the US. Patients were followed up until May 5, 2018. Subsequently, we randomly assigned participants to receive a management strategy that consisted of intra-articular glucocorticoid injections (n = 78) or physical therapy (n = 78), and the primary outcome was the difference in Western Ontario and McMaster Universities Osteoarthritis Index (WOMAC) total score at 1 year. We also collected WOMAC scores at 4, 6, and 26 weeks. Specific details of the treatment in each arm are reported in the published protocol and trial manuscript.^15,20^ Baseline characteristics of the cohort are described in Table 1.

**Table 1.** Descriptive and Demographic Variables of the 2 Groups at Baseline

Variable	All (n = 156)	Physical therapy (n = 78)	Glucocorticoid injection (n = 78)
Continuous variables, mean (SD)			
Age, y	56.1 (8.7)	56.3 (9.2)	56.0 (8.2)
BMI	31.5 (5.6)	31.4 (5.1)	31.6 (6.1)
Baseline WOMAC score (total score)	108.0 (44.7)	108.8 (47.1)	107.1 (42.4)
Duration of symptoms, mo	92.5 (107.2)	100.0 (122.7)	85.0 (89.2)
Categorical variables, No. (%)			
Sex			
Male	81 (51.9)	41 (52.6)	40 (51.3)
Female	75 (48.1)	37 (47.4)	38 (48.7)
Smoker	8 (5.1)	5 (6.4)	3 (3.8)
Beneficiary category			
Active duty^a^	41 (26.2)	20 (25.6)	21 (26.9)
Retired service member	54 (34.6)	28 (35.9)	26 (33.3)
Family member or dependent	61 (39.1)	29 (37.2)	32 (41.0)
Kellgren-Lawrence grade^b^			
1	6 (3.8)	5 (6.4)	1 (1.3)
2	68 (43.6)	26 (33.3)	42 (53.8)
3	59 (37.8)	34 (43.6)	25 (32.1)
4	23 (14.7)	13 (16.7)	10 (12.8)

Abbreviations: BMI, body mass index (calculated as weight in kilograms divided by height in meters squared); WOMAC, Western Ontario and McMaster Universities Arthritis Index.

^a^ Includes reserve or national guard on active duty.

^b^ Radiographic measures of severity are as follows: grade 1, doubtful joint space narrowing and possible osteophytic lipping; grade 2, definite osteophytes and possible joint space narrowing; grade 3, moderate multiple osteophytes, definite narrowing of joint space, and some sclerosis and possible deformity of bone ends; and grade 4, large osteophytes, marked narrowing of joint space, severe sclerosis, and definite deformity of bone ends.

### Costs

We used the Military Health System Data Repository (MDR) to source all costs of care. The MDR captures care for all outpatient and inpatient encounters, ancillary services (radiology and laboratory tests), and pharmacy transactions from military and civilian clinics where TRICARE (the health care system of the US Department of Defense) is the payer. The Military Health System is a closed, single-payer government health system, where typically less than 3% of patients younger than 65 years carry other health insurance.^22^ Patients in this study had no copayments, which allowed capture of all medical care that was sought by each participant. Exceptions could include care not covered and paid fully out-of-pocket, such as massage therapy or chiropractic services. Although there is no actual exchange of money for care in military facilities, each element of care is assigned a predetermined value and price by the US Defense Health Agency, which is reported in the MDR, to facilitate budgeting and business case analyses. We used these cost values for care in military facilities and costs actually paid by TRICARE for all care in-network facilities. We identified knee-related care through the use of the *International Classification of Diseases, Ninth Revision (ICD-9)* and *International Statistical Classification of Diseases and Related Health Problems, Tenth Revision (ICD-10)* diagnosis codes, as well as relevant procedures based on *Current Procedural Terminology* (*CPT*) codes. Both diagnoses and procedure codes used are listed in the eAppendix in the Supplement. Although care spanned a 6-year period, because participants were evenly randomized during this period, we did not make annual adjustments for costs. We created 2 cost outcomes: (1) total costs specifically for knee-related medical care and (2) total medical costs for any reason.

The adjusted generalized linear model with γ distribution and log link was used, controlling for age, sex, body mass index, smoking status, and Kellgren-Lawrence radiographic severity score.^23^ Estimated marginal means with 95% CIs were reported.

### Effectiveness

Effectiveness was measured using QALYs. We used an established mapping algorithm to generate EuroQol 5-Dimension (EQ-5D) health utilities from the WOMAC scores at all time points used in the trial (baseline, 4 weeks, 8 weeks, 26 weeks, and 1 year).^24^ The EQ-5D is used to assess the perceived limitations of an individual related to mobility, self-care, regular daily activities, discomfort or pain, and anxiety or depression.^25^ The utility score was calculated for each individual anchored from 0 (death) to 1 (perfect health). The QALY was calculated from estimated EQ-5D at the 1-year interval as follows: QALY = baseline EQ-5D + [(1-year EQ-5D − baseline EQ-5D)/2]. The effectiveness analysis was conducted using an adjusted generalized linear model, reporting estimated marginal means with 95% CIs.

### Cost-effectiveness

By comparing between treatment arms, the costs and QALYs were assessed at 1-year follow-up, using conventional decision rules and estimating incremental cost-effectiveness ratios (ICERs) as appropriate. The ICERs were calculated by dividing the difference in costs between both interventions by differences in effectiveness between both interventions. Although the effectiveness of physical therapy was superior in the original trial,^15^ a typical course of physical therapy may initially be more expensive and usually requires more resources (eg, additional visits). Therefore, we assessed whether an initial treatment choice of physical therapy could be cost-effective compared with an initial choice of glucocorticoid injection. The goal was to determine the incremental cost per effectiveness obtained. This goal was accomplished by calculating the incremental cost divided by the incremental effectiveness to derive the ICER.

The willingness-to-pay (WTP) estimates usually vary by country, with more developed countries often willing to pay more for improvements in QALYs. Although there is no universal consensus for what ICER threshold should be used in the US, we used the threshold of $100 000 per QALY because it is commonly used in the US, particularly with osteoarthritis populations,^26,27,28^ with compelling arguments to justify it as the minimum threshold.^29,30^ To express the most cost-effective strategy of the base case in US dollars, we used the net monetary benefit framework to demonstrate the net benefit of each strategy (net monetary benefit = [incremental effectiveness × WTP] – incremental cost). A positive value indicates the intervention is cost-effective at the given WTP level.

### Missing Data

Because this is a closed, single-payer government system, we assumed that lack of medical encounters within MDR indicated that the care did not happen, and therefore there were no missing health care utilization data. The MDR has a robust data validation system in which data are imported daily from more than 260 sources worldwide and missing elements are continuously validated across other data sources for 90 days before the validated variable is turned from raw to final+. We pulled all data 90 days after the last participant reached the 1-year mark to ensure final validated data for every individual. We assumed that missing data for the outcomes (WOMAC and subsequent EQ-5D estimation) were missing completely at random (MCAR) and tested this assumption using the Little MCAR test.

### Uncertainty

To manage the uncertainty associated with this calculation, we used a bootstrapping method composed of 1000 replications to obtain a 95% CI of the ICER. The resulting acceptability curve of these bootstrapped ICERs show the proportions of ICERs under the WTP level.

### Sensitivity Analysis

We planned a priori a series of sensitivity analyses. First, we considered 2 alternate mapping algorithms to establish health utilities from the WOMAC.^31,32^ For the third analysis, we considered only individuals for whom there were complete follow-up data at 1 year (complete case analysis; N = 150). The fourth and fifth analyses excluded patients who received treatment from the other arm (7 from the physical therapy arm received an injection; 14 from the injection arm received physical therapy).

### Statistical Analysis

The statistical significance level was set at 2-sided *P* < .05. The statistical analyses were conducted using Stata software, version 12 (StataCorp LLC).

## Results

A total of 156 participants (mean [SD] age, 56.1 [8.7] years; 81 [51.9%] male) were randomized 1:1 and followed up for 1 year. Six participants did not provide outcomes data at 1 year (5 in the injection group; 1 in the physical therapy group). For those individuals, we used multiple imputation (Markov chain Monte Carlo method, 20 iterations),^33^ using other follow-up time points as predictors in the imputation model. Every participant had at least 3 of the 5 total data time points. The Little MCAR test suggested that data were indeed missing at random (169.9, *P* = .51). Baseline variables and demographic characteristics of each group are given in Table 1. The impact inventory recommended for report with cost-effectiveness analysis 11 is found in eTable 1 in the Supplement. Four participants (all in the glucocorticoid injection group) had knee surgery during the 1-year follow-up. Most participants in the glucocorticoid injection group received more than 1 injection (mean, 2.6).

The difference in QALYs between groups was statistically significant, with participants in the physical therapy group gaining 0.08 more QALYs than those in the corticosteroid injection group (95% CI, 0.02-0.13; *P* = .03) (Table 2). Unadjusted model results are present in eTable 2 in the Supplement.

**Table 2.** Cost-effectiveness Analysis

Variable	Physical therapy (n = 78)	Corticosteroid injection (n = 78)	Difference	*P* value	Base case ICER, $	Bootstrapped ICER,^a^ $	Acceptability, %			INMB (WTP threshold of $100 000), $
							WTP threshold of $0	WTP threshold of $50 000	WTP threshold of $100 000	
QALYs (95% CI)^b^	0.76 (0.73 to 0.80)	0.69 (0.65 to 0.72)	0.08 (0.02 to 0.13)	.003	NA	NA	NA	NA	NA	NA
Knee-related cost (95% CI)^c^, $	2449 (1893 to 3004)	1834 (1454 to 2213)	615 (–34 to 1263)	.06	8103	8782 (1540 to 34 975)	0.3	98.5	99.2	6955
Total medical cost (95% CI)^c^, $	8921 (7208 to 10 634)	6776 (5476 to 8074)	2145 (12 to 4279)	.049	28 271	35 527 (1318 to 206 319)	2.0	82.7	97.2	5535

Abbreviations: ICER, incremental cost-effectiveness ratio; NA, not applicable; INMB, incremental net monetary benefit; QALYs, quality-adjusted life-years; WTP, willingness to pay.

^a^ The bootstrapping method with 1000 replications.

^b^ Generalized linear model controlling for age, body mass index, female sex, smoking status, and Kellgren-Lawrence radiographic severity score.

^c^ Generalized linear model with log link and γ distribution controlling for age, body mass index, female sex, smoking status, and Kellgren-Lawrence radiographic severity score.

Mean (SD) knee-related medical costs for the 1-year follow-up were similar between groups ($2113 [$4224] for glucocorticoid group; $2131 [$1015) for physical therapy) when unadjusted for any covariates (eTable 2 in the Supplement).^15^ These values included the trial intervention costs. When the costs were adjusted for age, body mass index, female sex, smoking status, and radiographic severity, however, participants in the physical therapy group spent an adjusted mean of $615 more than those in the glucocorticoid injection group, but the mean difference was not significant (95% CI, –$34 to $1263; *P* = .06) (Table 2). When it came to total medical costs for any reason, participants in the physical therapy group spent an adjusted mean of $2145 more than participants in the glucocorticoid injection group (95% CI, $12-$4279; *P* = .049) (Table 2).

### Cost-effectiveness

**Figure 1.**
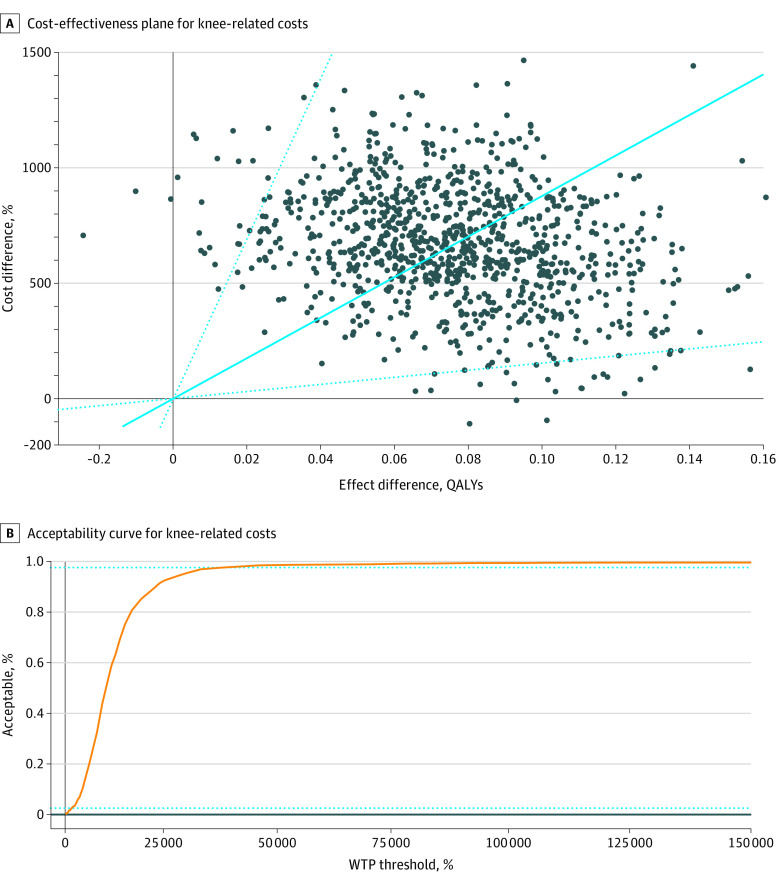
Cost-effectiveness Planes and Acceptability Curves for Knee-Related Costs Filled circles indicate the incremental cost-effectiveness ratios, with the solid blue line showing the mean incremental cost-effectiveness ratio. Dotted lines indicate 95% CIs. WTP indicates willingness to pay; QALY, quality-adjusted life-year.

**Figure 2.**
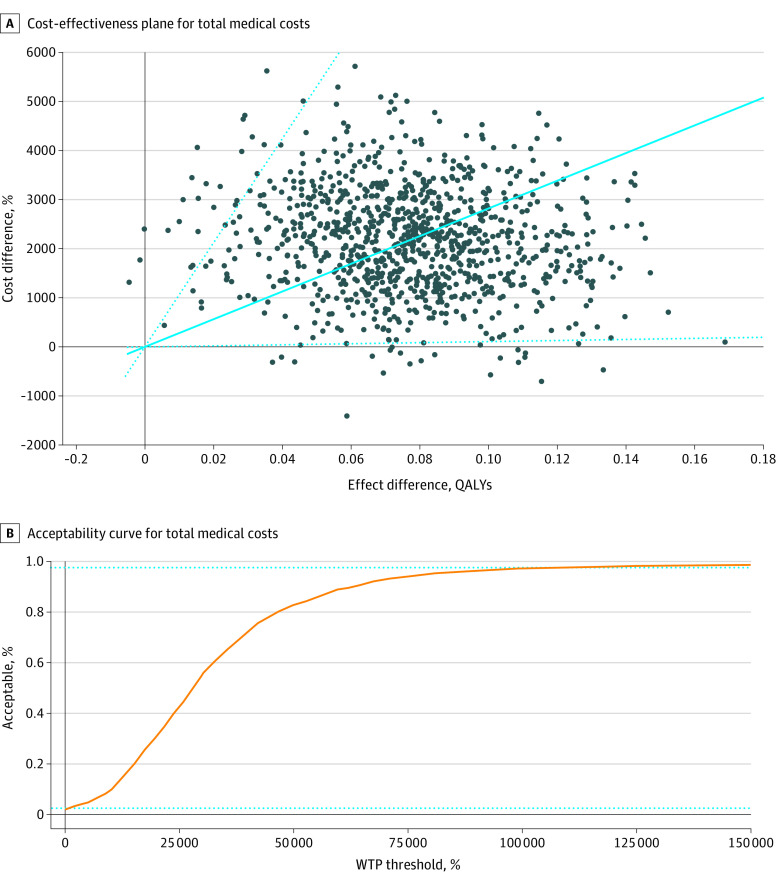
Cost-effectiveness Planes and Acceptability Curves for Total Medical Costs (Medical Care for Any Reason) Filled circles indicate the incremental cost-effectiveness ratios, with the solid blue line showing the mean incremental cost-effectiveness ratio. Dotted lines indicate 95% CIs. WTP indicates willingness to pay; QALY, quality-adjusted life-year.

**Figure 3.**
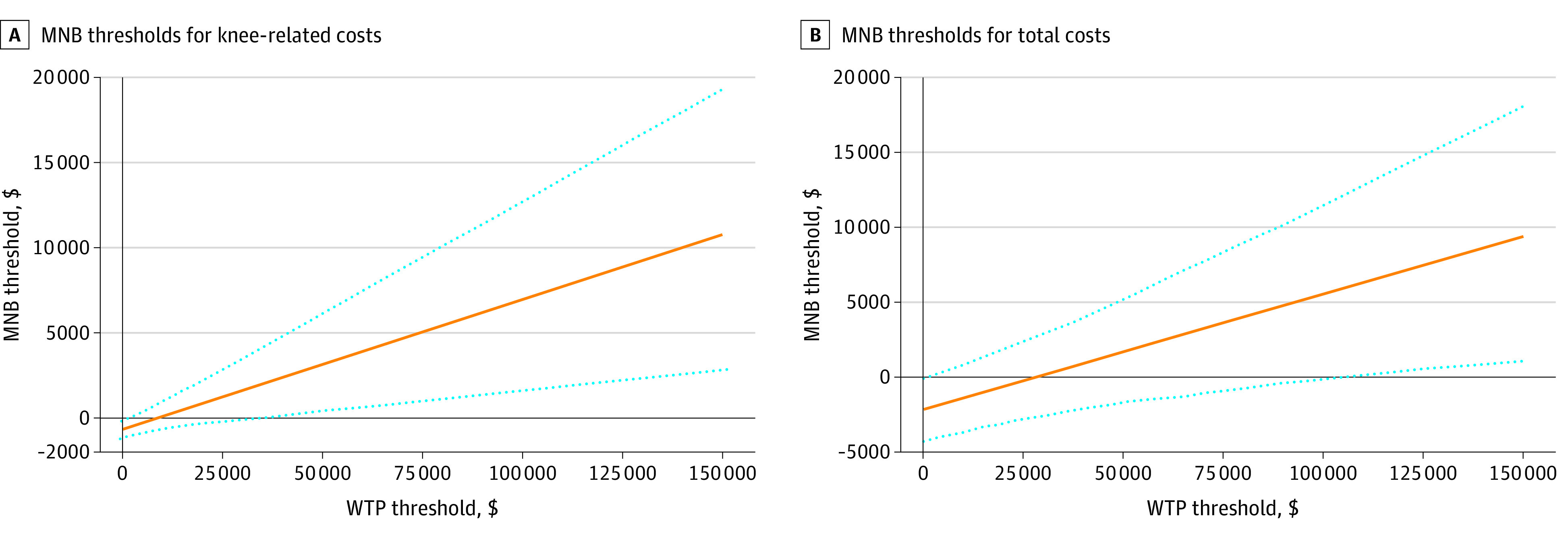
Monetary Net Benefit (MNB) Threshold Dotted lines indicate 95% CIs. WTP indicates willingness to pay.

Patients in the physical therapy group had a mean QALY of 0.076 more at a mean cost of $615 higher for knee-related medical costs than patients in the injection group, resulting in an ICER of $8103 per QALY. The ICER was $28 271 per QALY when it came to total medical costs for any reason. Both of these are much less than the common WTP thresholds of $50 000 and $100 000, suggesting that physical therapy is a cost-effective intervention despite higher initial costs than glucocorticoid injection. To address uncertainty, we obtained 1000 ICERs with bootstrapping that provided an ICER of $8782 per QALY in respect to knee-related medical costs and an ICER of $28 172 per QALY in respect to total medical costs for any reason (Figure 1). Our cost-effectiveness acceptability curve indicated that 99.2% of 1000 bootstrapped ICERs for knee-related costs and 97.2% for total medical costs for any reason were acceptable at the WTP threshold of $100 000 (Figure 2). The zero net monetary benefit threshold was at the WTP threshold $8103 for knee-related costs and $28 271 for total medical costs, indicating that physical therapy was cost-effective compared with glucocorticoid injection at any higher WTP level (Figure 3). The incremental net monetary benefit at the WTP threshold of $100 000 was $6955 for knee-related costs and $5535 for total medical costs (Figure 3).

### Sensitivity Analysis

After performing all 5 sensitivity analyses, the overall conclusions were unchanged (eTable 3 in the Supplement) The mean difference in QALYs significantly favored physical therapy in all models. The ICER for knee-related medical costs in these models ranged from $6079 to $31 665, all well below the WTP threshold of $100 000 (eTable 3 and eFigures 1-5 in the Supplement). The ICER for total medical costs was below the WTP threshold of $100 00 for all models (range, $24 241-$51 414) except for when using the EQ-5D mapping algorithm by Xie et al^25^ ($107 796; 95% CI, –$4446 to $412 770) (eTable 3 and eFigures 4 in the Supplement).^32^

## Discussion

This economic evaluation is the first study, to our knowledge, to assess the cost-effectiveness of physical therapy when considered as an alternative to glucocorticoid injection. The primary aim was to estimate the economic benefit of one common treatment for knee osteoarthritis over another, given the quality-of-life improvement and cost associated with each. This analysis suggests that an initial strategy of physical therapy is cost-effective at 1 year compared with an initial strategy of glucocorticoid injection, despite being more expensive than a glucocorticoid injection. Considering all the knee-related care costs during the entire 1-year follow-up, physical therapy resulted in an ICER of $8103 per QALY, which is well below the minimum WTP threshold of $100 000.

Costs, in both time and dollars, are likely relevant decision factors driving the use of each treatment. Physical therapy in this trial involved a typical course of 8 visits during 4 weeks, whereas the injection can be delivered during the initial consultation with guidance for 72 hours of reduced patient activity. For individuals who work or have other responsibilities, the idea of 1 injection could appear more favorable than 8 physical therapy sessions. Missing work to attend sessions can initially appear as a greater burden. However, the notion that 1 or more glucocorticoid injections will fix the problem is also not realistic. In this trial, most patients needed multiple injections (mean, 2.6). In our trial, 14 individuals in the injection group eventually went to physical therapy, and 4 individuals underwent surgery. None in the physical therapy group had surgery. This finding could explain some of the higher-than-expected costs in the injection group. Patients should be made aware of these outcomes when considering the 2 treatment options, as well as the short-term efficacy of a single glucocorticoid injection, typically 1 to 6 weeks,^34,35,36^ in addition to the associated risks.^10,11^ Patients may be told an injection can reduce acute pain to ensure that physical therapy is tolerable. The short-term improvement in pain was equal for both interventions in our trial. Prior studies^12,13^ have found that adding corticosteroid injections to physical therapy or exercise therapy does not improve outcomes beyond the use of these interventions alone.

Other studies^26,27,37,38^ have assessed the cost-effectiveness of physical therapy for knee osteoarthritis. Bove and colleagues^26^ compared 4 physical therapy regimens: exercise and manual therapy or exercise alone, each with and without booster treatment sessions. Booster sessions typically consist of 1 to 3 visits that occur several months after the initial physical therapy episode of care to provide additional treatment, check exercises, and provide further reassurance and encouragement. Either treatment group was more cost-effective if it included the booster sessions, despite the additional cost incurred with the extra visits. In similar fashion, our trial also included an option for a booster session for all patients in both the physical therapy and corticosteroid injection arms. Silva et al^27^ found a 3-year physical activity program for nonactive patients with knee osteoarthritis to be cost-effective when considering long-term improvements in QALYs and reductions in diabetes and cardiovascular disease severity. The ICER in the study by Bove et al^26^ was $12 900 per QALY gained and was $16 100 per QALY gained for Silva et al.^27^ In a study in New Zealand,^37^ exercise therapy and manual physical therapy were both cost-effective compared with usual care, but the combination of both was not. For the group that received a combination of both, investigators noted time restraints that likely reduced the dosing and potentially the effect size. Those findings are noteworthy, although that study had a different comparator than was used in our study, given that the physical therapy program in our study integrated manual techniques with exercise therapy, and patients received an adequate dose of both. Another study^38^ in the UK found 12 sessions of physical therapy to be cost-effective during a 30-month period when compared with usual care.

After a series of robust sensitivity analyses based on various assumptions, our results remained unchanged. Physical therapy remained cost-effective compared with glucocorticoid injection in every sensitivity model associated with knee-related medical costs. When considering total medical costs for any reason, all models except for one found physical therapy to be cost-effective. Although this was only 1 of 6 models, total medical costs for any reason is likely not ideal for measuring cost-effectiveness because there is the potential for many additional confounders when considering total health costs for any reason (compared with only knee-related costs). Many of these factors are outside the ability of the research team to control in the analysis (eg, comorbidities or unrelated operations and procedures).

### Limitations

We used a single-payer government health system, which can limit the generalizability of these findings to other US health systems. For example, individuals seeking care in military hospitals and clinics do not usually have copayments, and direct cost would not be a barrier to seeking care. Costs of physical therapy can vary widely; therefore, different health plans could affect the cost-effectiveness of this intervention. Our focus was on the health care perspective, and as such, we did not capture data from the societal perspective; however, it is likely that the generalizability of results that focus on the societal perspective would also have been limited to this particular setting. Although we had outcomes from the WOMAC available at 5 time points, the costs represented a single value of costs expensed during the entire year and therefore could not be attributed to each of the time points individually. Finally, we used a mapping algorithm to generate EQ-5D values from the WOMAC, and although we conducted multiple sensitivity analyses using several other prominent mapping algorithms, it is possible that results would be different if QALYs were available directly from the EQ-5D. These results need validation in other settings.

## Conclusions

A course of physical therapy was a cost-effective intervention compared with a course of glucocorticoid injections for patients enrolled in this trial. Clinicians should consider that although the initial cost of delivering physical therapy may be slightly higher than an initial glucocorticoid injection, the mean improvement in QALYs at 1 year may also be greater with physical therapy. The combination of superior long-term outcomes, cost-effectiveness, and low risk with physical therapy as provided in this clinical trial provides an additional perspective to inform shared decision-making about value-based case between health care practitioners and patients.

## Notes

Supplement. **eAppendix.** International Classification of Diseases (ICD) Codes–9th and 10th Edition and Current Procedural Terminology (CPT) Codes Associated With Knee Osteoarthritis and Knee-Related Care
eReferences
**eTable 1.** Impact Inventory **eTable 2.** Models Without Accounting for Any Covariates (Unadjusted Models) **eTable 3.** Sensitivity Analyses for Primary Cost-Effectiveness Analysis **eFigure 1.** Histogram of QALY Scores in Each Intervention Group **eFigure 2.** Histogram of Knee-Related Costs Scores in Each Intervention Group **eFigure 3.** Histogram of Total Costs Scores in Each Intervention Group **eFigure 4.** Cost-effectiveness Planes and Acceptability Curves for the Sensitivity Analyses
