# Supplementary material for: Cost-effectiveness of Physical Therapy vs Intra-articular Glucocorticoid Injection for Knee Osteoarthritis: A Secondary Analysis From a Randomized Clinical Trial
Source: JAMA Netw Open. 2022 Jan 24;5(1):e2142709. doi: 10.1001/jamanetworkopen.2021.42709 (PMC8787617; doi:10.1001/jamanetworkopen.2021.42709)
Supplement: Supplement. — eAppendix. International Classification of Diseases (ICD) Codes–9th and 10th Edition and Current Procedural Terminology (CPT) Codes Associated With Knee Osteoarthritis and Knee-Related Care eReferences eTable 1. Impact Inventory eTable 2. Models Without Accounting for Any Covariates (Unadjusted Models) eTable 3. Sensitivity Analyses for Primary Cost-Effectiveness Analysis eFigure 1. Histogram of QALY Scores in Each Intervention Group eFigure 2. Histogram of Knee-Related Costs Scores in Each Intervention Group eFigure 3. Histogram of Total Costs Scores in Each Intervention Group eFigure 4. Cost-effectiveness Planes and Acceptability Curves for the Sensitivity Analyses [file jamanetwopen-e2142709-s001.pdf]

Supplementary Online Content

Rhon DI, Kim M, Asche CV, Allison SC, Allen CS, Deyle GD. Cost-effectiveness of physical therapy vs intra-articular glucocorticoid injection for knee osteoarthritis: a secondary analysis from a randomized clinical trial. *JAMA Netw Open*. 2022;5(1):e2142709. doi:10.1001/jamanetworkopen.2021.42709

eAppendix. International Classification of Diseases (ICD) Codes–9th and 10th Edition and Current Procedural Terminology (CPT) Codes Associated With Knee Osteoarthritis and Knee-Related Care
eReferences
eTable 1. Impact Inventory
eTable 2. Models Without Accounting for Any Covariates (Unadjusted Models)
eTable 3. Sensitivity Analyses for Primary Cost-Effectiveness Analysis
eFigure 1. Histogram of QALY Scores in Each Intervention Group
eFigure 2. Histogram of Knee-Related Costs Scores in Each Intervention Group
eFigure 3. Histogram of Total Costs Scores in Each Intervention Group
eFigure 4. Cost-effectiveness Planes and Acceptability Curves for the Sensitivity Analyses

This supplementary material has been provided by the authors to give readers additional information about their work.

© 2022 Rhon DI et al. *JAMA Network Open.*

eAppendix. International Classification of Diseases (ICD) Codes – 9th and 10th Edition and Current Procedural Terminology (CPT) Codes Associated With Knee Osteoarthritis and Knee-Related Care

Note: The list of codes is sensitive, but majority of these knee-related codes were not present in this cohort.

oa_knee
**Flag list includes:

ICD9 Codes	Description
715.16	Osteoarthrosis, localized, primary, lower leg
715.26	Osteoarthrosis, localized, secondary, lower leg
715.36	Osteoarthrosis, localized, not specified whether primary or secondary, lower leg
715.96	Osteoarthrosis, unspecified whether generalized or localized, lower leg

ICD10 Codes	Description
M17.0	Bilateral primary osteoarthritis of knee
M17.10	Unilateral primary osteoarthritis, unspecified knee
M17.11	Unilateral primary osteoarthritis, right knee
M17.12	Unilateral primary osteoarthritis, left knee
M17.2	Bilateral post-traumatic osteoarthritis of knee
M17.30	Unilateral post-traumatic osteoarthritis, unspecified knee
M17.31	Unilateral post-traumatic osteoarthritis, right knee
M17.32	Unilateral post-traumatic osteoarthritis, left knee
M17.4	Other bilateral secondary osteoarthritis of knee
M17.5	Other unilateral secondary osteoarthritis of knee
M17.9	Osteoarthritis of knee, unspecified
M25.761	Osteophyte, right knee
M25.762	Osteophyte, left knee
M25.769	Osteophyte, unspecified knee**

knee_related_care
**Flag list includes:

ICD9 Code	Description
716.16	Traumatic arthropathy, lower leg
716.46	Transient arthropathy, lower leg
716.66	Unspecified monoarthritis, lower leg
716.86	Other specified arthropathy, lower leg
716.96	Arthropathy, unspecified Arthropathy, unspecified, lower leg
717.0	Old bucket handle tear of medial meniscus
717.1	Derangement of anterior horn of medial meniscus
717.2	Derangement of posterior horn of medial meniscus
717.3	Other and unspecified derangement of medial meniscus
717.40	Derangement of lateral meniscus, unspecified
717.41	Bucket handle tear of lateral meniscus

© 2022 Rhon DI et al. *JAMA Network Open.*

717.42	Derangement of anterior horn of lateral meniscus
717.43	Derangement of posterior horn of lateral meniscus
717.49	Other derangement of lateral meniscus
717.5	Derangement of meniscus, not elsewhere classified
717.6	Loose body in knee
717.7	Chondromalacia of patella
717.81	Old disruption of lateral collateral ligament
717.82	Old disruption of medial collateral ligament
717.83	Old disruption of anterior cruciate ligament
717.84	Old disruption of posterior cruciate ligament
717.85	Old disruption of other ligaments of knee
717.89	Other internal derangement of knee
717.9	Unspecified internal derangement of knee
718.26	Pathological dislocation of joint, lower leg
718.36	Recurrent dislocation of joint, lower leg
718.56	Ankylosis of joint, lower leg
718.86	Other joint derangement, not elsewhere classified, lower leg
719.06	Effusion of joint, lower leg
719.16	Hemarthrosis, lower leg
719.26	Villonodular synovitis, lower leg
719.46	Pain in joint, lower leg
719.56	Stiffness of joint, not elsewhere classified, lower leg
719.66	Other symptoms referable to joint, lower leg
719.86	Other specified disorders of joint, lower leg
719.96	Unspecified disorder of joint, lower leg
726.60	Enthesopathy of knee, unspecified
726.61	Pes anserinus tendinitis or bursitis
726.62	Tibial collateral ligament bursitis
726.63	Fibular collateral ligament bursitis
726.64	Patellar tendinitis
726.65	Prepatellar bursitis
726.69	Other enthesopathy of knee
727.51	Synovial cyst of popliteal space
727.65	Nontraumatic rupture of quadriceps tendon
727.66	Nontraumatic rupture of patellar tendon
727.83	Plica syndrome
729.31	Hypertrophy of fat pad, knee
836.0	Tear of medial cartilage or meniscus of knee, current
836.1	Tear of lateral cartilage or meniscus of knee, current
836.2	Other tear of cartilage or meniscus of knee, current
836.3	Dislocation of patella, closed
836.4	Dislocation of patella, open
836.50	Dislocation of knee, unspecified, closed
836.51	Anterior dislocation of tibia, proximal end, closed
836.52	Posterior dislocation of tibia, proximal end, closed
836.53	Medial dislocation of tibia, proximal end, closed

© 2022 Rhon DI et al. *JAMA Network Open.*

836.54	Lateral dislocation of tibia, proximal end, closed
836.59	Other dislocation of knee, closed
836.60	Dislocation of knee, unspecified, open
836.61	Anterior dislocation of tibia, proximal end, open
836.62	Posterior dislocation of tibia, proximal end, open
836.63	Medial dislocation of tibia, proximal end, open
836.64	Lateral dislocation of tibia, proximal end, open
836.69	Other dislocation of knee, open
844.0	Sprain of lateral collateral ligament of knee
8844.1	Sprain of medial collateral ligament of knee
844.2	Sprain of cruciate ligament of knee
844.3	Sprain of tibiofibular (joint) (ligament) superior, of knee
844.8	Sprains and strains of other specified sites of knee and leg
844.9	Sprains and strains of unspecified site of knee and leg

ICD10 Code	Description
M12.261	Villonodular synovitis (pigmented), right knee
M12.262	Villonodular synovitis (pigmented), left knee
M12.269	Villonodular synovitis (pigmented), unspecified knee
M12.561	Traumatic arthropathy, right knee
M12.562	Traumatic arthropathy, left knee
M12.569	Traumatic arthropathy, unspecified knee
M12.861	Other specific arthropathies, not elsewhere classified, right knee
M12.862	Other specific arthropathies, not elsewhere classified, left knee
M12.869	Other specific arthropathies, not elsewhere classified, unspecified knee
M13161	Monoarthritis, not elsewhere classified, right knee
M13162	Monoarthritis, not elsewhere classified, left knee
M13169	Monoarthritis, not elsewhere classified, unspecified knee
M13861	Other specified arthritis, right knee
M13862	Other specified arthritis, left knee
M13869	Other specified arthritis, unspecified knee
M14861	Arthropathies in other specified diseases classified elsewhere, right knee
M14862	Arthropathies in other specified diseases classified elsewhere, left knee
M14869	Arthropathies in other specified diseases classified elsewhere, unspecified knee
M22	Disorders of Patella
M23	Internal Derangement of Knee
M24361	Pathological dislocation of right knee, not elsewhere classified
M24362	Pathological dislocation of left knee, not elsewhere classified
M24369	Pathological dislocation of unspecified knee, not elsewhere classified
M24461	Recurrent dislocation, right knee
M24462	Recurrent dislocation, left knee
M24469	Recurrent dislocation, unspecified knee

© 2022 Rhon DI et al. *JAMA Network Open.*

M24661	Ankylosis, right knee
M24662	Ankylosis, left knee
M24669	Ankylosis, unspecified knee
M2506	Hemarthrosis knee
M25161	Fistula, right knee
M25162	Fistula, left knee
M25169	Fistula, unspecified knee
M25261	Flail joint, right knee
M25262	Flail joint, left knee
M25269	Flail joint, unspecified knee
M25361	Other instability, right knee
M25362	Other instability, left knee
M25369	Other instability, unspecified knee
M25461	Effusion, right knee
M25462	Effusion, left knee
M25469	Effusion, unspecified knee
M25561	Pain in right knee
M25562	Pain in left knee
M25569	Pain in unspecified knee
M25661	Stiffness of right knee, not elsewhere classified
M25662	Stiffness of left knee, not elsewhere classified
M25669	Stiffness of unspecified knee, not elsewhere classified
M25861	Other specified joint disorders, right knee
M25862	Other specified joint disorders, left knee
M25869	Other specified joint disorders, unspecified knee
M65161	Other infective (teno)synovitis, right knee
M65162	Other infective (teno)synovitis, left knee
M65169	Other infective (teno)synovitis, unspecified knee
M660	Rupture of popliteal cyst
M6736	Transient synovitis, rknee
M6746	Ganglion, knee
M675	Plica syndrome, knee
M6786	Other specified disorders of synovium, knee
M704	Prepatellar bursitis, knee
M7050	Other bursitis of knee, knee
M712	Synovial cyst of popliteal space [Baker], knee
M7146	Calcium deposit in bursa, knee
M71561	Other bursitis, not elsewhere classified, right knee
M71562	Other bursitis, not elsewhere classified, left knee
M71569	Other bursitis, not elsewhere classified, unspecified knee
M71861	Other specified bursopathies, right knee
M71862	Other specified bursopathies, left knee
M71869	Other specified bursopathies, unspecified knee
M7630	Iliotibial band syndrome, unspecified leg
M7631	Iliotibial band syndrome, right leg
M7632	Iliotibial band syndrome, left leg

© 2022 Rhon DI et al. *JAMA Network Open.*

M7640	Tibial collateral bursitis [Pellegrini-Stieda], unspecified leg
M7641	Tibial collateral bursitis [Pellegrini-Stieda], right leg
M7642	Tibial collateral bursitis [Pellegrini-Stieda], left leg
M7650	Patellar tendinitis, unspecified knee
M7651	Patellar tendinitis, right knee
M7652	Patellar tendinitis, left knee
M794	Hypertrophy of (infrapatellar) fat pad
M94261	Chondromalacia, right knee
M94262	Chondromalacia, left knee
M94269	Chondromalacia, unspecified knee

anesthesia_knee CPT

Flag includes: Anesthesia for Procedures on the Knee and Popliteal Area CPT Code range 01320-01444

Thigh-knee_surgical_procedures CPT

Surgical Procedures of the Femur (thigh region) and knee joint CPT Code range 27301-27599

knee_arthroscopy CPT

Flag includes CPT Codes 29850-29889; G0289

xray_knee CPT

Flag includes:

73560 Radiologic examination, knee; one or two views

73562 Radiologic examination, knee; three views

73564 Radiologic examination, knee; complete, four or more views

73565 Radiologic examination, knee; both knees, standing, anteroposterior

arthrogram_knee CPT

Flag includes: 73580 Radiologic examination, knee, arthrography, radiological supervision and interpretation

© 2022 Rhon DI et al. *JAMA Network Open.*

eReferences

1. Sanders GD, Neumann PJ, Basu A, *et al.* Recommendations for Conduct, Methodological Practices, and Reporting of Cost-effectiveness Analyses: Second Panel on Cost-Effectiveness in Health and Medicine. *JAMA* 2016;**316**:1093–103.
2. Xie F, Pullenayegum EM, Li S-C, *et al.* Use of a disease-specific instrument in economic evaluations: mapping WOMAC onto the EQ-5D utility index. *Value Health* 2010;**13**:873–8.
3. Barton GR, Sach TH, Jenkinson C, *et al.* Do estimates of cost-utility based on the EQ-5D differ from those based on the mapping of utility scores? *Health Qual Life Outcomes* 2008;**6**:51.

© 2022 Rhon DI et al. *JAMA Network Open.*

eTable 1. Impact Inventory[1]

Sector	Type of Impact (list category within each sector with unit of measure if relevant)[a]	Included in This Reference Case Analysis From…Perspective?		Notes on Sources of Evidence
		Health Care Sector	Societal	
**Formal Health Care Sector**				
Health	**Health outcomes (effects)**			
	Longevity effects	☐	☐	
	Health-related quality-of-life effects	X	☐	
	Other health effects (eg, adverse events and secondary transmissions of infections)	X	☐	
	**Medical costs**			
	Paid for by third-party payers	X	☐	
	Paid for by patients out-of-pocket	☐	☐	
	Future related medical costs (payers and patients)/	X	☐	
	Future unrelated medical costs (payers and patients)	X	☐	
**Informal Health Care Sector**				
**Health**	Patient-time costs	NA	☐	
	Unpaid caregiver-time costs	NA	☐	
	Transportation costs	NA	☐	
**Non-Health Care Sectors (with examples of possible items)**				
**Productivity**	Labor market earnings lost	NA	☐	
	Cost of unpaid lost productivity due to illness	NA	☐	
	Cost of uncompensated household production[b]	NA	☐	
**Consumption**	Future consumption unrelated to health	NA	☐	
**Social Services**	Cost of social services as part of intervention	NA	☐	
**Legal of Criminal Justice**	Number of crimes related to intervention	NA	☐	
	Cost of crimes related to intervention	NA	☐	
**Education**	Impact of intervention on educational achievement of population	NA	☐	
**Housing**	Cost of intervention on home improvements (eg, removing lead paint)	NA	☐	
**Environment**	Production of toxic waste pollution by intervention	NA	☐	
**Other (specify)**	Other impacts	NA	☐	

eTable 2. Models Without Accounting for Any Covariates (Uunadjusted Models)

	Physical Therapy	Steroid Injection	Mean Between Group Difference	ICER (base case)	ICER (bootstrapped)[A]	% of Acceptability WTP			INMB WTP = 100,000
						0	50,000	100,000	
QALY (95% CI)[B]	0.761 (0.724, 0.798)	0.691 (0.653, 0.727)	0.071 (0.018, 0.123) (p=0.008)						
Knee-related cost (95% CI[B]	$2131 ($1443, $2818)	$2113 ($1431, $2795)	$18 (-$950, $986) (p=0.063)	$256	$314 (-$14,453, $23,826)	44.6%	98.7%	99.2%	$7098
Total medical cost (95% CI)[B]	$8946 ($7002, $10,888)	$6786 ($5312, $8260)	$2,159 (-$279, $4597) (p=0.083)	$30,600	$30,915 (-$2644, $141,419)	3.6%	75.4%	94.6%	$4905

ICER=Incremental Cost-Effectiveness Ratio; WTP = Willingness to Pay; INMB = Incremental Net Monetary Benefit

[A]The bootstrapping method with 1,000 replications

[B]Generalized linear model without controlling for any other variables

© 2022 Rhon DI et al. *JAMA Network Open.*

eTable 3. Sensitivity Analyses for Primary Cost-Effectiveness Analysis

A. Model using alternate EQ-5D Mapping Algorithm by Xie et al.
B. Model using alternate EQ-5D Mapping Algorithm by Barton et al.
C. Model removing 6 subjects that did not have 1-year data
D. Model removing 7 patients originally randomized to the Physical Therapy group that received Corticosteroid Injection (outside the purview of study care) during the 1-year period of surveillance, after their initial Physical Therapy treatment.
E. Model removing 14 patients originally randomized to Corticosteroid Injection group that received Physical Therapy (outside the purview of study care) during the 1-year period of surveillance, after their initial Injection.

	Physical Therapy	Steroid Injection	Mean Between Group Difference	ICER (base case)	ICER (bootstrapped)[A]	% of Acceptability WTP			INMB WTP = 100,000
						0	50,000	100,000	
**A. Using alternate EQ-5D Mapping Algorithm by Xie et al.**									
QALY (95% CI)[B]	0.783 (0.773, 0.791)	0.763 (0.754, 0.772)	0.019 (0.007, 0.032) (p=0.002)						
Knee-related cost (95% CI[C]	$2449 ($1893, $3004)	$1834 ($1454, $2213)	$615 (-$34, $1263) (p=0.063)	$31,665	$33,047 ($6689, $113,305)	0.5%	77.5%	96.2%	$1322
Total medical cost (95% CI)[C]	$8921 ($7208, $10,634)	$6776 ($5476, $8074)	$2,145 ($12, $4279) (p=0.049)	$110,482	$107,796 (-$4446, $412,770)	2.8%	16.1%	45.4%	-$155
**B. Using alternate EQ-5D Mapping Tool by Barton et al.**									
QALY (95% CI)[B]	0.665 (0.641, 0.689)	0.619 (0.594, 0.643)	0.046 (-0.011, 0.081) (p=0.01)						
Knee-related cost (95% CI)[C]	$2449 ($1893, $3004)	$1834 ($1454, $2213)	$615 (-$34, $1263) (p=0.063)	$13,316	$13,531 ($2287, -$91,329)	0.6%	95.0%	97.6%	$4105
Total medical cost (95% CI)[C]	$8921 ($7208, $10,634)	$6776 ($5476, $8074)	$2,145 ($12, $4279) (p=0.049)	$46,460	$46,567 ($1882, -$244,341)	2.0%	55.8%	86.6%	$2491

© 2022 Rhon DI et al. *JAMA Network Open.*

**C. Model removing 6 subjects that did not have primary outcome data at 1 year**									
QALY (95% CI)[B]	0.765 (0.729, 0.800)	0.695 (0.659, 0.731)	0.070 (p=0.008) (0.018, 0.121)						
Knee-related cost (95% CI)[C]	$2355 ($1879, $2829)	$1619 ($1300, $1936)	$736 ($168, $1303) (p=0.011)	$10,562	$10,691 ($3360, $50,286)	0.1%	97.3%	99.0%	$6370
Total medical cost (95% CI)[C]	$8386 ($6882, $9890)	$6215 ($5069, $7359)	$2,172 ($295, $4047) (p=0.023)	$31,171	$30,849 ($6076, $157,584)	0.8%	80.1%	93.8%	$4899
**D. Removal of 7 patients from physical therapy group that later received glucocorticoid injection**									
QALY (95% CI)[B]	0.773 (0735, 0.810)	0.689 (0.653, 0.724)	0.084 (0.032, 0.136) (p=0.002)						
Knee-related cost (95% CI)[C]	$2350 ($1793, $2907)	$1839 ($1459, $2218)	$511 (p=0.120) (-$134, $1156)	$6079	$6396 ($653, $23,454)	1.5%	99.6%	99.7%	$7893
Total medical cost (95% CI)[C]	$8866 ($7038, $10692)	$6827 ($5477, $8176)	$2,038 (p=0.078) (-$226, $4303)	$24,241	$24,517 (-$1826, $95,134)	3.8%	88.1%	97.6%	$6376
**E. Removal of 14 patients from corticosteroid injection group that later received physical therapy**									
QALY (95% CI)[B]	0.765 (0.731, 0.798)	0.703 (0.655, 0.740)	0.062 (0.011, 0.113) (p=0.017)						
Knee-related cost (95% CI)[C]	$2221 ($1824, $2617)	$1326 ($1071, $1581)	$895 (p<0.001) ($425, $1363)	$14,409	$14,915 ($6405, -$173,534)	0.0%	93.1%	96.5%	$5274
Total medical cost (95% CI)[C]	$8846 ($7167, $10524)	$5699 ($4501, $6896)	$3,147 (p=0.002) ($1107, $5187)	$50,684	$51,414 ($15,422, $358,387)	0.2%	48.9%	84.7%	$3043

ICER=Incremental Cost-Effectiveness Ratio; WTP = Willingness to Pay; INMB = Incremental Net Monetary Benefit

[A]The bootstrapping method with 1,000 replications

[B]Generalized linear model controlling age, BMI, female, smoker and Kellgren-Lawrence radiographic severity score

[C]Generalized linear model with log link and gamma distribution controlling age, BMI, female, smoker and Kellgren-Lawrence radiographic severity score

© 2022 Rhon DI et al. *JAMA Network Open.*

eFigure 1. Histogram of QALY Scores in Each Intervention Group

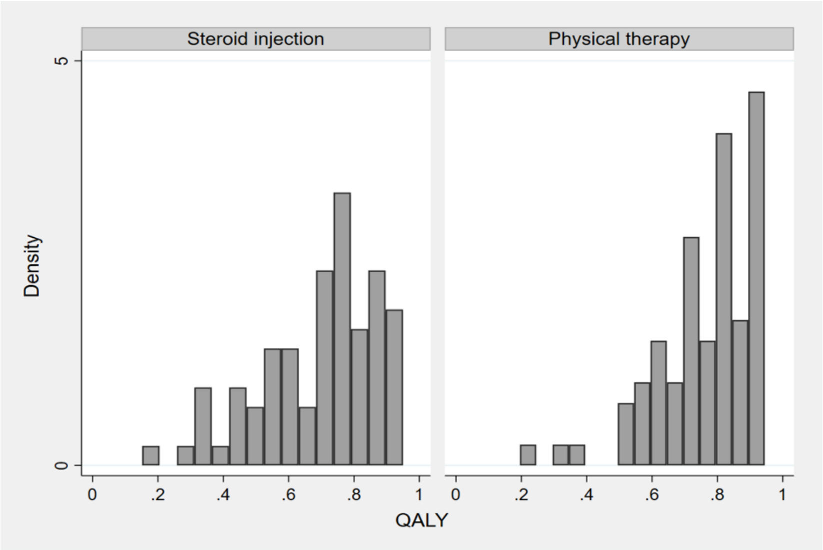

© 2022 Rhon DI et al. *JAMA Network Open.*

eFigure 2. Histogram of Knee-Related Costs Scores in Each Intervention Group

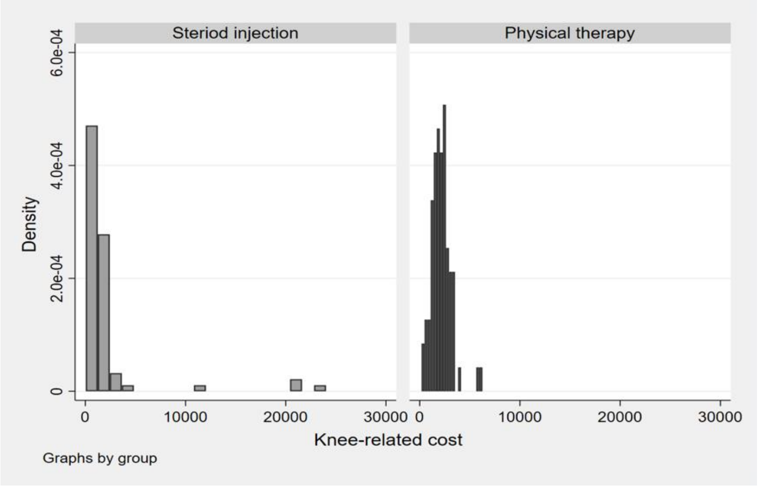

© 2022 Rhon DI et al. *JAMA Network Open.*

eFigure 3. Histogram of Total Costs Scores in Each Intervention Group

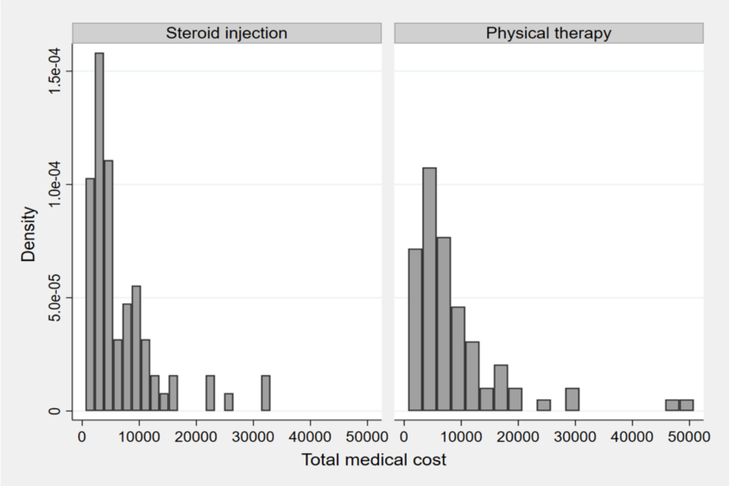

© 2022 Rhon DI et al. *JAMA Network Open.*

eFigure 4. Cost-effectiveness Planes and Acceptability Curves for the Sensitivity Analyses

Sensitivity Analysis A: Cost effectiveness planes and acceptability curve
(Using an alternate EQ-5D Mapping Algorithm by Xie et al.)[1]

Knee-related costs (SA1, SA2)

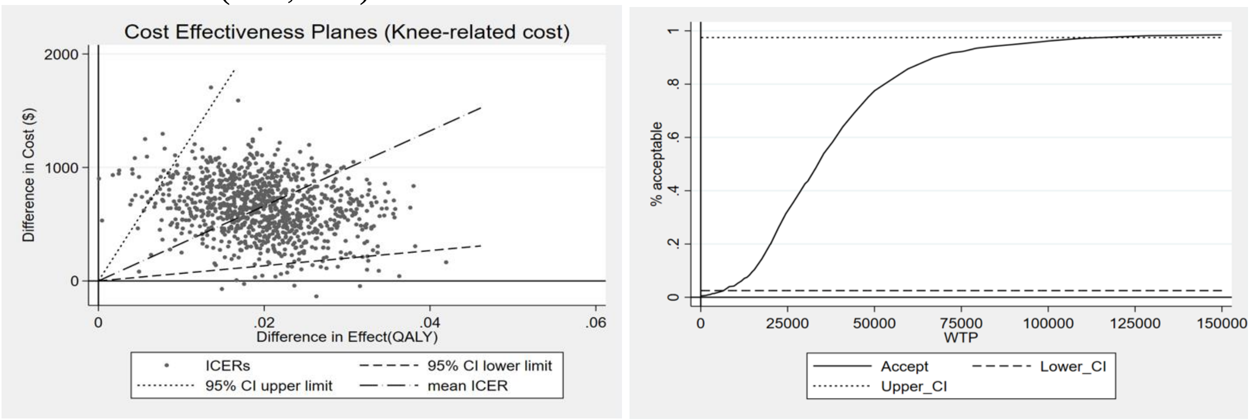

Total medical costs (SA3, SA4)

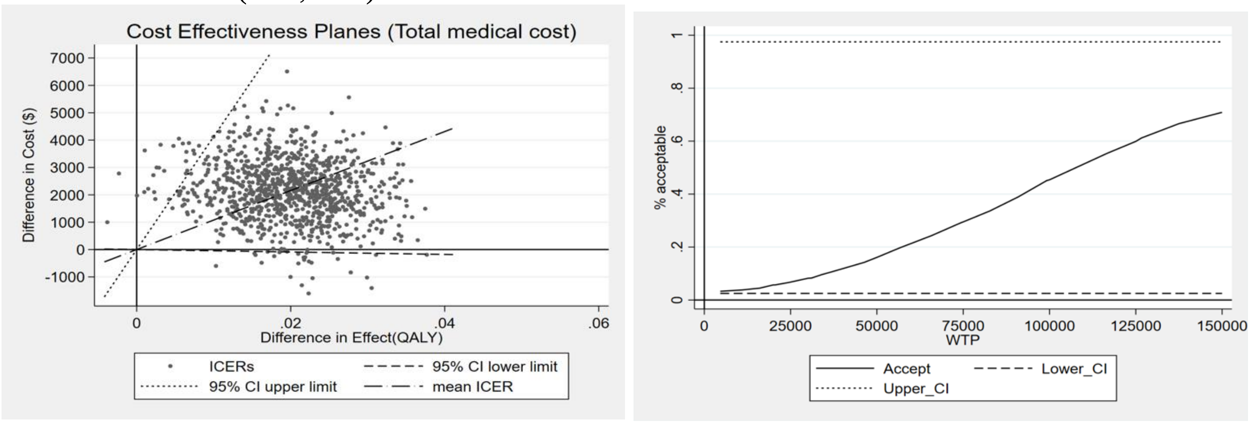

© 2022 Rhon DI et al. *JAMA Network Open.*

Sensitivity Analysis B: Cost effectiveness planes and acceptability curve
(Model using an alternate EQ-5D Mapping Tool by Barton et al.)[2]

Knee-related costs (SB1, SB2)

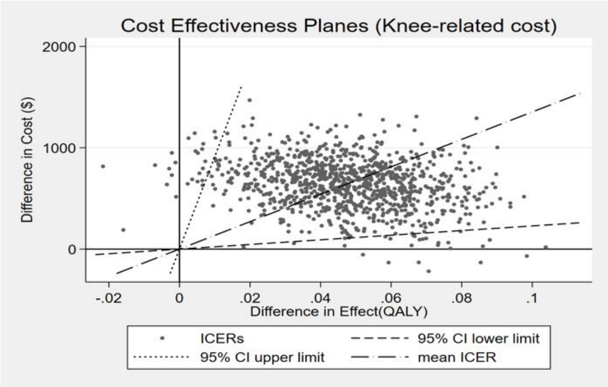

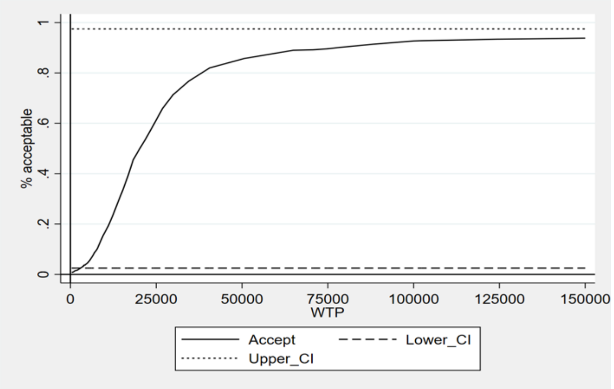

Total medical costs (SB3, SB4)

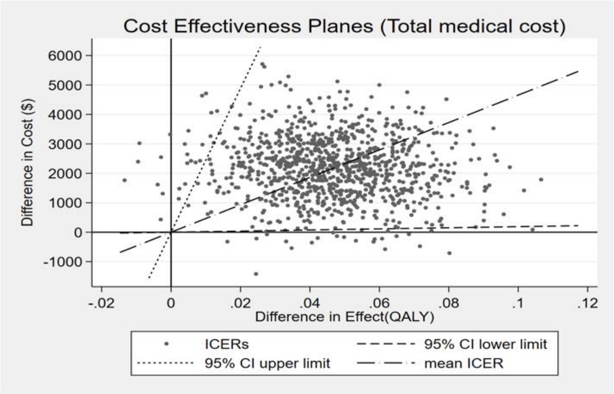

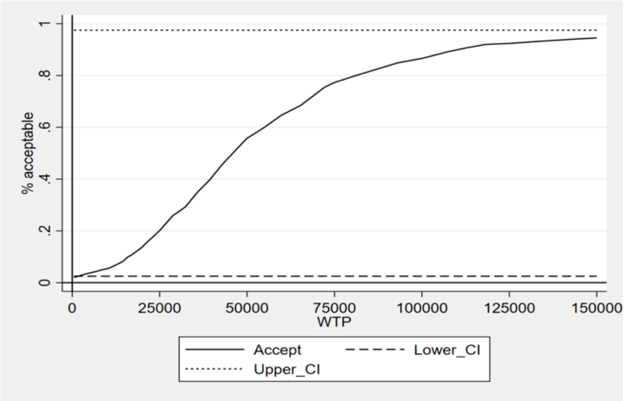

© 2022 Rhon DI et al. *JAMA Network Open.*

Sensitivity Analysis C: Cost effectiveness planes and acceptability curve

(Model removing 6 subjects that did not have primary outcome data at 1 year)

Knee-related costs (SC1, SC2)

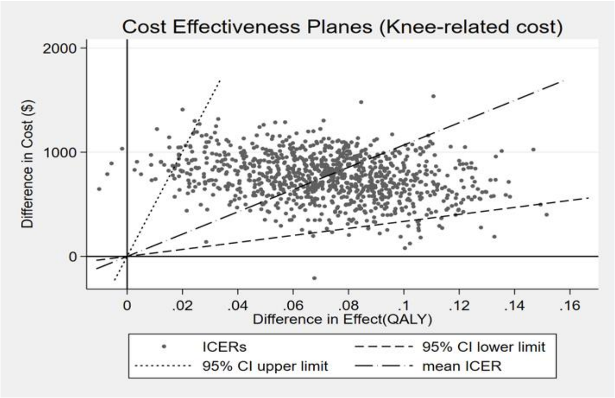

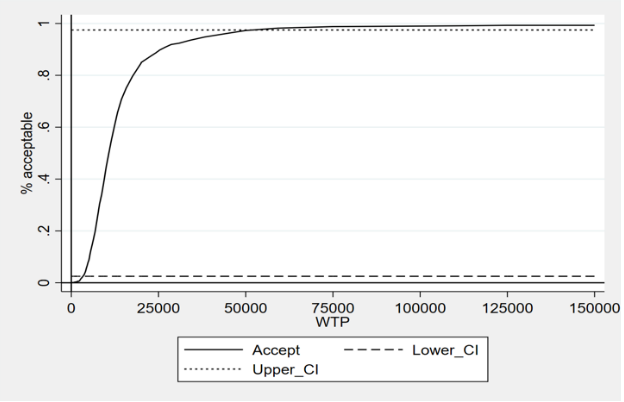

Total medical costs (SC3, SC4)

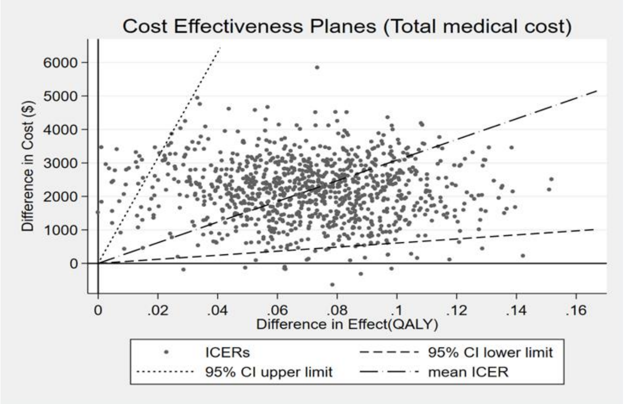

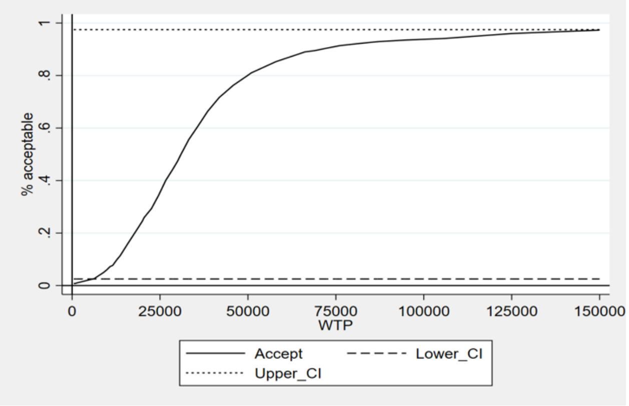

© 2022 Rhon DI et al. *JAMA Network Open.*

Sensitivity Analysis D: Cost effectiveness planes and acceptability curve
(Model removing 7 patients from physical therapy group that later received glucocorticoid injection)

Knee-related costs (SD1, SD2)

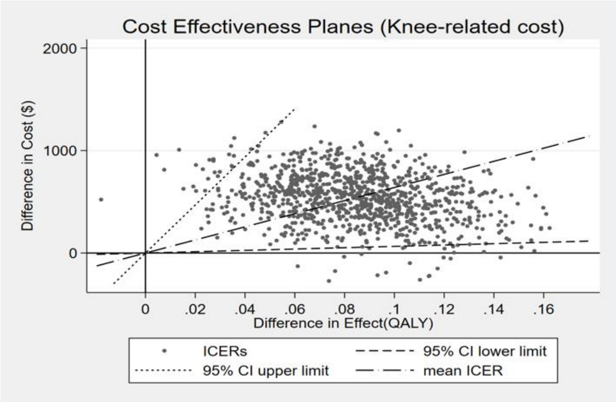

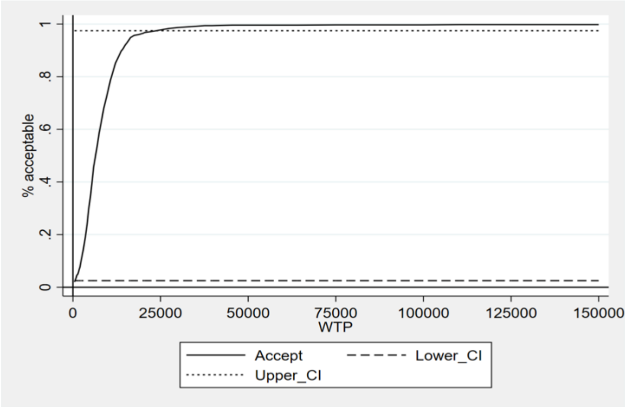

Total medical costs (SD3, SD4)

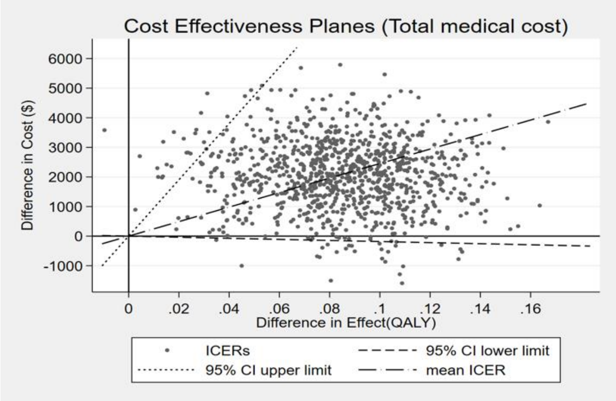

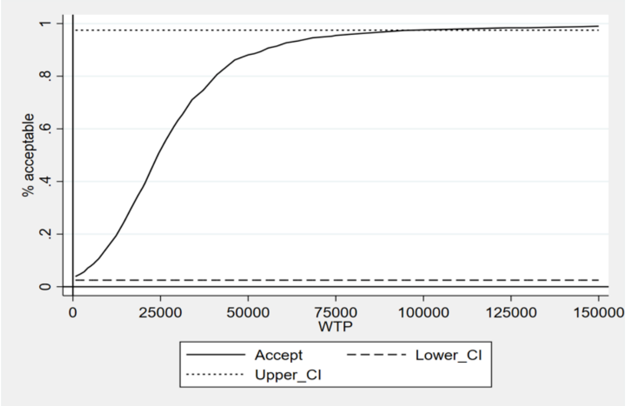

© 2022 Rhon DI et al. *JAMA Network Open.*

Sensitivity Analysis E: Cost effectiveness planes and acceptability curve
(Model removing 14 patients from corticosteroid injection group that later received physical therapy)

Knee-related costs (SE1, SE2)

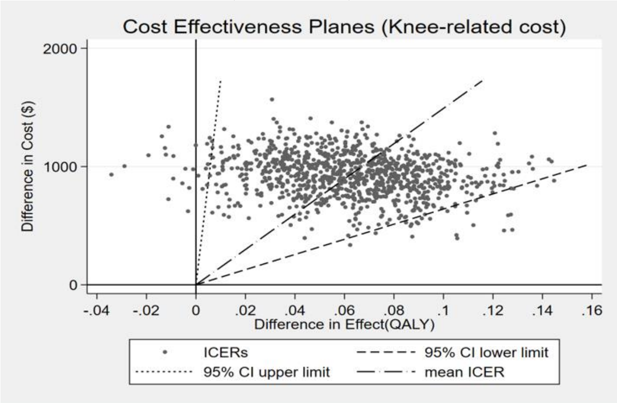

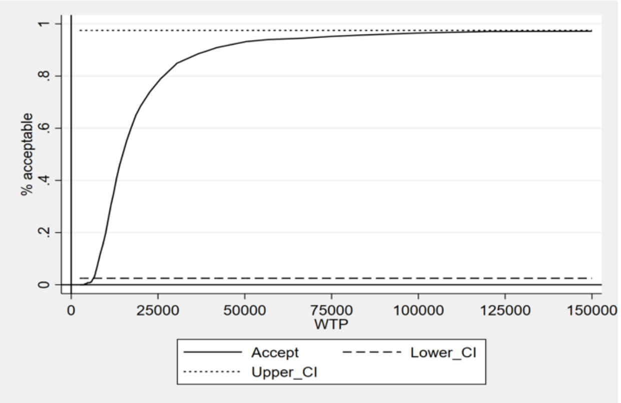

Total medical costs (SE3, SE4)

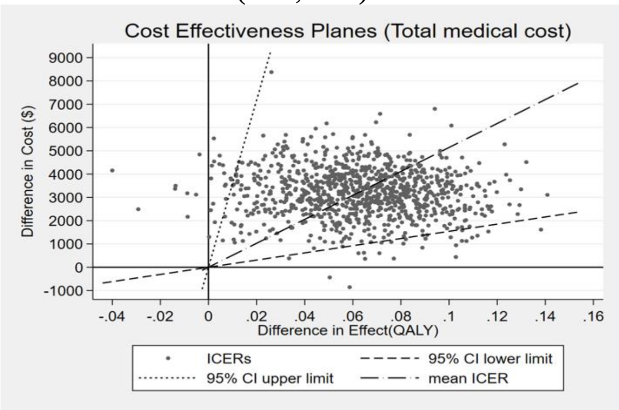

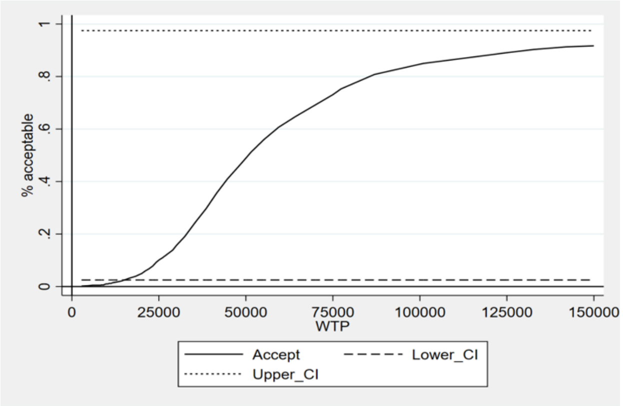

© 2022 Rhon DI et al. *JAMA Network Open.*

© 2022 Rhon DI et al. *JAMA Network Open.*
